# A Microfluidic-Based Sensing Platform for Rapid Quality Control on Target Cells from Bioreactors

**DOI:** 10.3390/s24227329

**Published:** 2024-11-16

**Authors:** Alessia Foscarini, Fabio Romano, Valeria Garzarelli, Antonio Turco, Alessandro Paolo Bramanti, Iolena Tarantini, Francesco Ferrara, Paolo Visconti, Giuseppe Gigli, Maria Serena Chiriacò

**Affiliations:** 1 CNR Nanotec Institute of Nanotechnology, Via Monteroni, 73100 Lecce, Italy; alessia.foscarini@nanotec.cnr.it (A.F.); valeria.garzarelli@nanotec.cnr.it (V.G.); antonio.turco@nanotec.cnr.it (A.T.); iolena.tarantini@unisalento.it (I.T.); giuseppe.gigli@unisalento.it (G.G.); mariaserena.chiriaco@nanotec.cnr.it (M.S.C.); 2 Department of Experimental Medicine, University of Salento, 73100 Lecce, Italy; 3 Department of Innovation Engineering, University of Salento, Via per Monteroni, Building ‘O’, 73100 Lecce, Italy; fabioromano9198@gmail.com (F.R.); alessandro.bramanti@st.com (A.P.B.); paolo.visconti@unisalento.it (P.V.); 4 Department of Mathematics and Physics, E. De Giorgi, University of Salento, Via per Arnesano, 73100 Lecce, Italy; 5 STMicroelectronics srl, c/o Campus Ecotekne, Via per Monteroni, 165, 73100 Lecce, Italy

**Keywords:** lab-on-chip, cell detection, capacitive sensor, on-chip sensor, CAR-T quality control

## Abstract

We investigated the design and characterization of a Lab-On-a-Chip (LoC) cell detection system primarily designed to support immunotherapy in cancer treatment. Immunotherapy uses Chimeric Antigen Receptors (CARs) and T Cell Receptors (TCRs) to fight cancer, engineering the response of the immune system. In recent years, it has emerged as a promising strategy for personalized cancer treatment. However, it requires bioreactor-based cell culture expansion and manual quality control (QC) of the modified cells, which is time-consuming, labour-intensive, and prone to errors. The miniaturized LoC device for automated QC demonstrated here is simple, has a low cost, and is reliable. Its final target is to become one of the building blocks of an LoC for immunotherapy, which would take the place of present labs and manual procedures to the benefit of throughput and affordability. The core of the system is a commercial, on-chip-integrated capacitive sensor managed by a microcontroller capable of sensing cells as accurately measured charge variations. The hardware is based on standardized components, which makes it suitable for mass manufacturing. Moreover, unlike in other cell detection solutions, no external AC source is required. The device has been characterized with a cell line model selectively labelled with gold nanoparticles to simulate its future use in bioreactors in which labelling can apply to successfully engineered CAR-T-cells. Experiments were run both in the air—free drop with no microfluidics—and in the channel, where the fluid volume was considerably lower than in the drop. The device showed good sensitivity even with a low number of cells—around 120, compared with the 10^7^ to 10^8^ needed per kilogram of body weight—which is desirable for a good outcome of the expansion process. Since cell detection is needed in several contexts other than immunotherapy, the usefulness of this LoC goes potentially beyond the scope considered here.

## 1. Introduction

Immunotherapy leverages the training of immune effectors, such as T lymphocytes, against cancer cells. Genetically engineered T cells can express Chimeric Antigen Receptors (CARs) and T Cell Receptors (TCRs), designed to recognize cancer cells by specific molecular markers on their surface. In this way, personalized treatments are developed and tailored to the specific needs of each patient [1,2].

While holding great potential, current immunotherapy approaches require enclosed bioreactors for CAR-T cell culture expansion and manual control, which is time-consuming, labour-intensive, costly, and susceptible to human error [3,4]. Moreover, bioreactors are inaccessible during the expansion phase, which prevents the real-time monitoring of the process. Thus, quality control (QC), which is necessary to check whether and to what extent the genetic modification has been effective, can be carried out only at the end of the process, just before reinfusing the cells into the patient [5,6].

Typically, 10^7^–10^8^ CAR-T cells per kilogram of body weight are required to consider an efficient reinfusion dose in patients. A low amount of modified cells or a low rate of proliferation means that something in the process of lymphocyte engineering or in cell culture conditions is wrong (the nutrient amount, possible contaminations, pH or temperature, just to list some elements to be considered) [7,8]. Moreover, the subpopulation of interest only differs from the other cells in the bioreactor by a single membrane antigen, retaining their shape and dimensions. 

In addition, the production of CAR-T cells has to follow strict rules according to the so-called current good manufacturing practises (CGMPs) with the aim of keeping the high standards of quality and safety required by clinical grade products. In particular, being a “living drug”, quality control includes the inspection of raw materials used in production, the controls on the process, and a final test on finished products [7].

Most of the tests needed for quality control are time-consuming and require a large amount of the sample (which is precious for patient reinfusion) as well as heavy instrumentation. They include immunophenotypic analysis [9] and cytofluorimetric assays confirmed by PCR [10]. An innovative approach has been proposed based on quality-by-design (QbD) to systematically investigate the impact of critical process parameters (CPPs) during the expansion step on the critical quality attributes (CQAs) and the quality of CAR-T cells culturing [11].

Anyway, possible failures cannot be detected in real-time, which may lead to the unacceptable loss of resources and waste of time, especially if the patient needs urgent therapy. A tool for decision-making in a real-time manner is then crucial. Lab-On-a-Chip (LoC) devices can be the answer. Extensively explored during the last decades, LoCs just miniaturize and automate lab operations, traditionally performed by human operators, integrating microfluidics and electronics. In addition, LoC analysis techniques generally require minimal quantities of biological samples and reagents (down to a few pico-litres), which results in cheaper tests and reduced invasiveness [12,13,14]. This could be key for an integrated QC function, which would check in real time the outcome of the cell modification based on the presence of a specific membrane antigen on the surface of CAR-T cells [15,16]. A small analyte volume would, in fact, minimize the subtraction from the final fluid volume—or maximize the quantity of personalized drug eventually reinfused in the patient.

Efforts have already been made to scale the detection protocols of biomarkers of contamination or T-cell activation down to a few microliter volumes spilled out from expansion chambers [8,17,18]. Such limited amounts of fluids are easily manipulated, leveraging state-of-the-art fabrication and microfluidic techniques [12,19].

In this study, we demonstrate a Lab-On-a-Chip (LoC) cell detector that combines simple microfluidics with a commercial capacitive sensor enhanced by a customized electric interface to balance cost and performance effectively. Originally designed for consumer applications, particularly touch interfaces, this commercial sensor can measure charge (or capacitive) variations (QVARs). Here, the sensor is connected to a microfabricated sensing unit, which includes a pair of microelectrodes integrated with a microfluidic focusing channel. The microelectrodes at the bottom of the fluidic channel are externally connected to the sensor. As cells flow through the channel, which has a volume of approximately 600 nL, they are selectively marked for the presence of a specific membrane antigen. Cells expressing the antigen also carry a gold nanoparticle, which induces charge variations detected by the sensor. After appropriate data post-processing, the system can estimate the percentage of modified cells.

Tested with cancer cells as a proof of concept, the system can be employed with virtually any type of labelled cells, identifying different subsets of cells for applications in immunotherapy and beyond [20]. Its simplicity, low cost, and sensitivity meet the requirements for an integrated QC real-time device with minimal impact on the overall sample volume.

## 2. Materials and Methods

Polymethyl Methacrylate (PMMA) substrates with optical surface quality (measured surface roughness (Ra) < 5 nm) (Vistacryl CQ; Vista Optics, Ltd., Widnes, Cheshire, WA8 0RP, UK) and glass substrates (Visiontek System Ltd., Upton Chester Cheshire, UK) were used to fabricate the microfluidic and the sensing module, respectively. The assembled devices were 25 mm^2^ squares, 3 mm and 1 mm thick in the upper and bottom layers, respectively. A thin layer of Polydimethylsiloxane (PDMS) (Sylgard™ 184, The Dow Company Inc., Midland, MI, USA) Clear Silicone Elastomer and a thermal treatment were used to bond the two materials together. A phosphate-buffered saline (PBS) solution, ethanol and Dulbecco’s modified Eagle medium (DMEM) were purchased from Sigma Aldrich (St. Louis, MO, USA) at the maximum purity grade. Immortalized prostate cancer cells (PC3) were cultured in standard conditions (DMEM supplemented with 10% fetal bovine serum and 1% penicillin/streptomycin, 37 °C, 5% CO_2_). Anti-EpCAM antibody (Sigma Aldrich)-functionalized gold nanoparticles (AuNPs) (Sigma Aldrich) were used to label PC3 (LbPC3) cells as appropriate. The anti-EpCAM mouse monoclonal antibodies specifically recognized human EpCAM expressed on the surface of epithelial cells. To confirm the labelling of cells with AuNPs, a secondary FITC antibody (Sigma Aldrich) was used to bind and amplify the signal of the primary EpCAM antibody so that fluorescent cells could be observed (Supplementary Materials Figure S1b).

## 3. Methods

### 3.1. Device Fabrication

**Figure 1:**
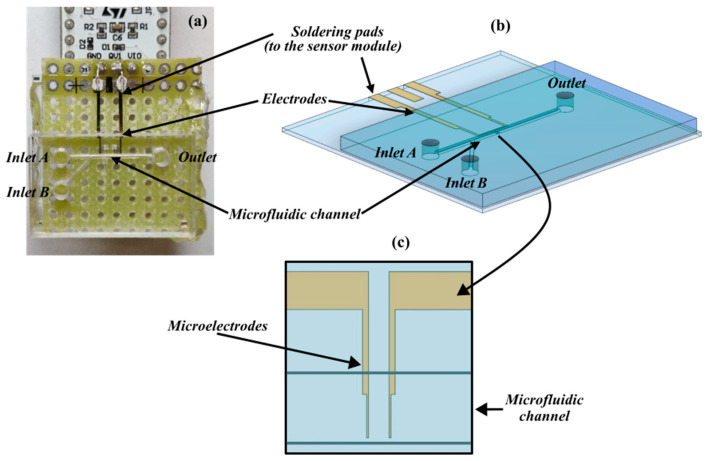
Components of the detection platform. (**a**) Overlook of the device. (**b**) Three-dimensional model rendering the microfluidics interface aligned with the sensing module. (**c**) Detail of the sensing area with the microelectrodes embedded into the microfluidic channel.

The detection platform, as shown in Figure 1a, includes a microfluidic module realized on a PMMA substrate by a micromilling machine (Minitech Machinery, Norcross, GA, USA) and bonded on a glass substrate (holding the sensing module) through a PDMS layer. The channel is Y-shaped to include two inlets through which the medium is injected into cells and the carrier fluid (culture medium or saline solution) to focus cells. The main arm of the channel (straight path) is 10 mm long, 300 μm wide, and 200 μm deep.

Flow control through the two inlets (Elvesys, Paris, France) allows the overall flow rate to be modulated and separation dynamics to be realized. The micropumping system used was equipped with an OB1 base module, four MkIII+ channels for the pressure controller, and four flow sensors. The sample was injected in the microfluidic channel at a velocity of 7 μL/min; in this way, the entire volume of the channel (around 600 nL) transits between the electrodes for little more than 5 s.

Two gold microelectrodes (Figure 1b) were fabricated by laser lithography (DWL66, Heidelberg Instruments, Heidelberg, Germany) on a glass substrate and aligned to be arranged on the bottom of the microfluidic channel. The portion of the sensing element in the channel included two finger electrodes separated by a gap around 40 μm (Figure 1c) in the same order of magnitude as the cell’s diameter (10 to 15 μm) for better sensitivity. At the other end, they were pad-shaped and micro-welded to the integrated circuit board of the sensor (Figure 1a) for QVAR detection.

### 3.2. Electronics

**Figure 2:**
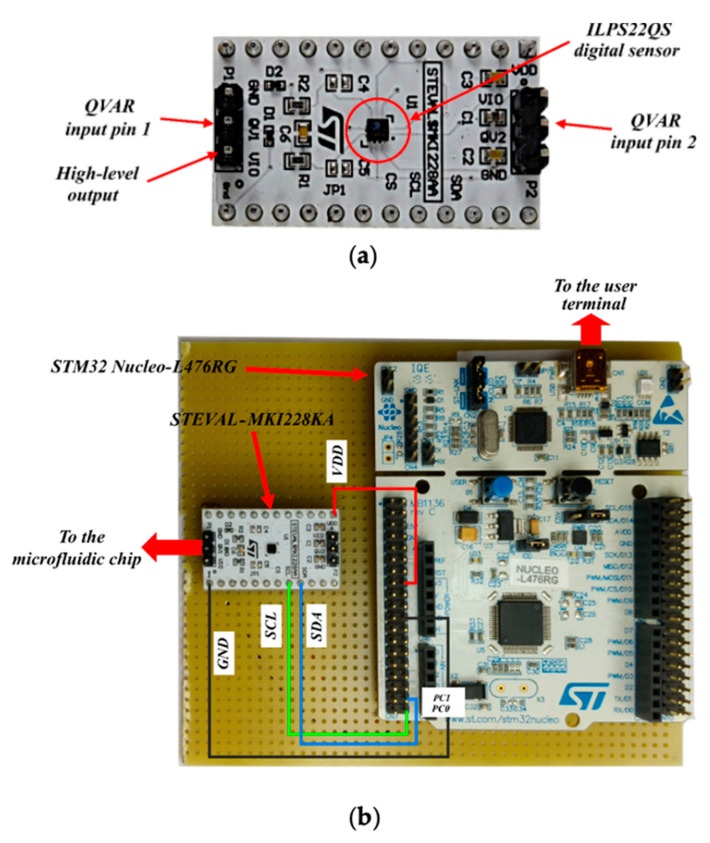
(**a**) The ILPS22QS sensor. (**b**) Overlook of the electronic part, including the STEVAL-MKI228KA sensor module and the STM32 Nucleo-L476RG board.

The electronic core of the system is a commercial capacitive sensor—ILPS22QS by STMicroelectronics [21]—which is a general-purpose device for pressure, temperature and capacitive measurements (charge to voltage conversion, down to a 10 µV sensitivity). The sensor amplifies and digitalizes the output voltage in a 24-bit signed string, which is stored in the output registers that become readable from an external PC through a microcontroller. Charge variation measurements (QVARs) are at the core of this work. The voltage is measured between a pair of contacts (pins protruding from the integrated circuit package, Figure 2a), which, in the system demonstrated here, are soldered to the two microelectrodes on the channel floor to sense the electromagnetic perturbation caused by the cell flow within the inter-electrodic space).

The sensor interfaces with the outer environment through some additional circuitry mounted on a sensor module board (STEVAL-MKI228KA by [22]). The whole system, in turn, is locally managed by a microcontroller (STM32L476RG by STMicroelectronics (Genf, Switzerland) [23]), mounted on a rapid prototyping board (STM32 Nucleo-L476RG by STMicroelectronics [24]) for programming, readout, and for interfacing with an outer PC (Figure 2b).

### 3.3. Software

The software developed for this system includes the firmware in the STM32L476RG microcontroller (MCU), designed to control the ILPS22QS sensor at the hardware level, and a MATLAB© (Mathworks, version: Matlab 2023b) user interface—an overview is given in the Supplementary Figure S2 diagram.

The firmware (developed on the STM32Cube platform and written in C) initializes the sensor, sets the Output Data Rate (ODR, 200 Hz, the maximum allowed), reads the voltage values and sends them time-stamped to the outer PC. The sensor and MCU communicate through the I2C interface, while transmission to the user interface occurs through the UART protocol.

The MATLAB© platform user interface logs and post-processes the data. The data are represented in real-time as an animated line and can be post-processed at the end of an established acquisition period.

## 4. Results

### 4.1. Preliminary Static Characterization

**Figure 3:**
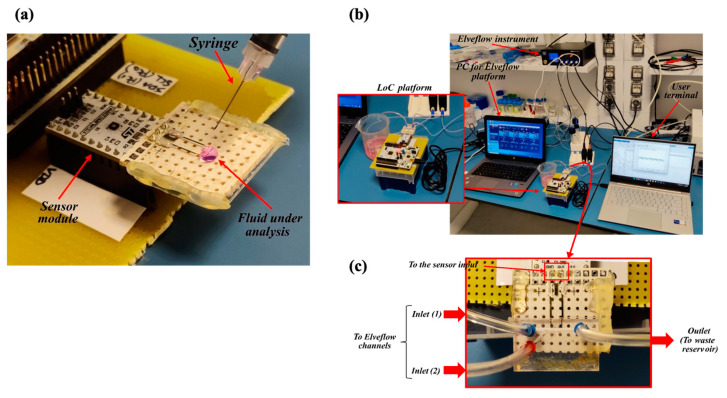
Benchtop configuration for the LoC testing. (**a**) First configuration for static testing without microfluidics. (**b**) The second configuration emulating the QC in the bioreactor. (**c**) Detail of the microfluidic connection.

Although the final purpose of the LoC is to detect cells flowing through the microchannel, a preliminary characterization of the capacitive sensor was performed prior to assembling the microfluidics. With this aim, an experiment was performed using only the sensing module and depositing a drop of solution directly on the electrodes’ surface, as shown in Figure 3a. For this preliminary test, 50 µL of the fluid to be investigated was used.

The drop was deposited slightly before instant 0 of the acquisition period, and the curves were cleaned of artifacts often observed at the deposition time. Unless otherwise mentioned, measurements were repeated at least three times and curves are shown to represent the average measurement.

Initially, cell-free fluids were used, namely phosphate-buffered saline (PBS), deionized water (DIW), ethanol, and Dulbecco’s modified Eagle medium (DMEM). As expected, the amplitudes of the traces (Supplementary Figure S3) correlated with the conductivities of the fluids, confirming the proper functioning of the system.

**Figure 4:**
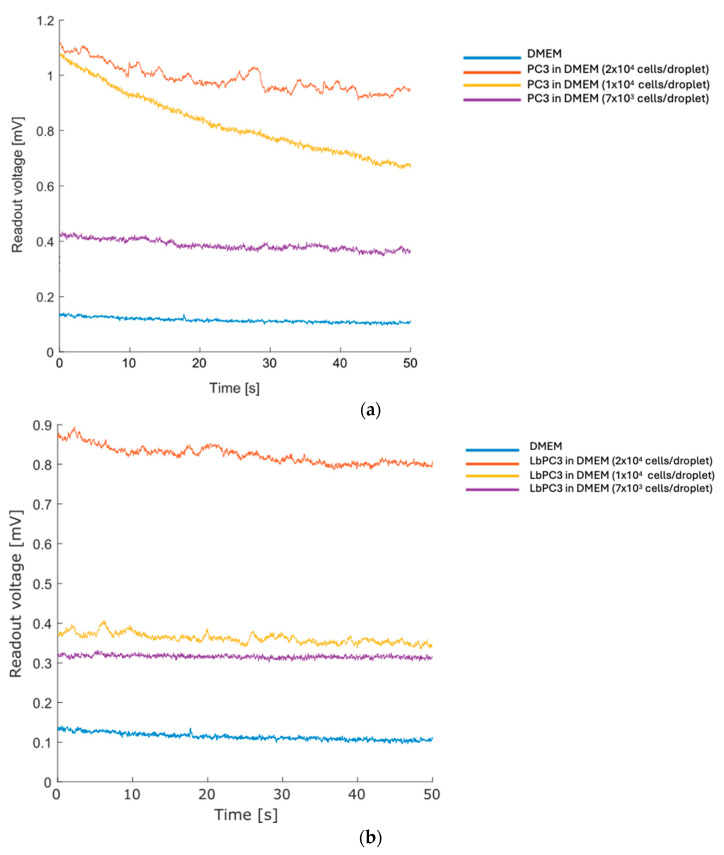
Prostate cancer cells serial dilution recording. (**a**) Curves of decreasing PC3 concentrations recorded by the deposition of cell droplets resuspended in DMEM. (**b**) Curves of decreasing EpCAM–gold nanoparticle-labelled PC3 (LbPC3) cell concentrations recorded by the deposition of cell droplets resuspended in DMEM.

Subsequently, a suspension of human prostate cancer cells (PC3) in DMEM was examined at varying concentrations, starting from an initial concentration of 4 × 10^5^ cells/mL through serial dilutions until 1/3 of the initial concentration was used in cell-free culture medium. After recording a baseline on DMEM (average voltage: 0.11 mV), a 50 µL drop for each of the three different cell concentrations was analyzed (Figure 4a). The sensor clearly distinguishes dilutions down to the lowest concentration tested. Considering the volume employed, the estimated number of cells per drop was around 2 × 10^4^, 1 × 10^4^, and 7 × 10^3^ cells at the respective tested dilutions.

Finally, LbPC3 cells (PC3 labelled with gold nanoparticles, AuNPs) were tested—see the Materials section for the labelling details. The labelling is specific, leveraging the coupling between the EpCAM antigen on the PC3 membrane and the functionalization of the nanoparticles with the Anti-EpCAM antibody. This mimics a condition in which some cells are labelled since they belong to a subpopulation; in the final device, these were modified. The tests were conducted at the same cell concentrations performed in the previous tests, and the results are summarized in Figure 4b.

Some curves show a slight decrease, probably due to the rearrangement of the drop. The trend is moderate, making the calculation of the average value meaningful.

**Figure 5:**
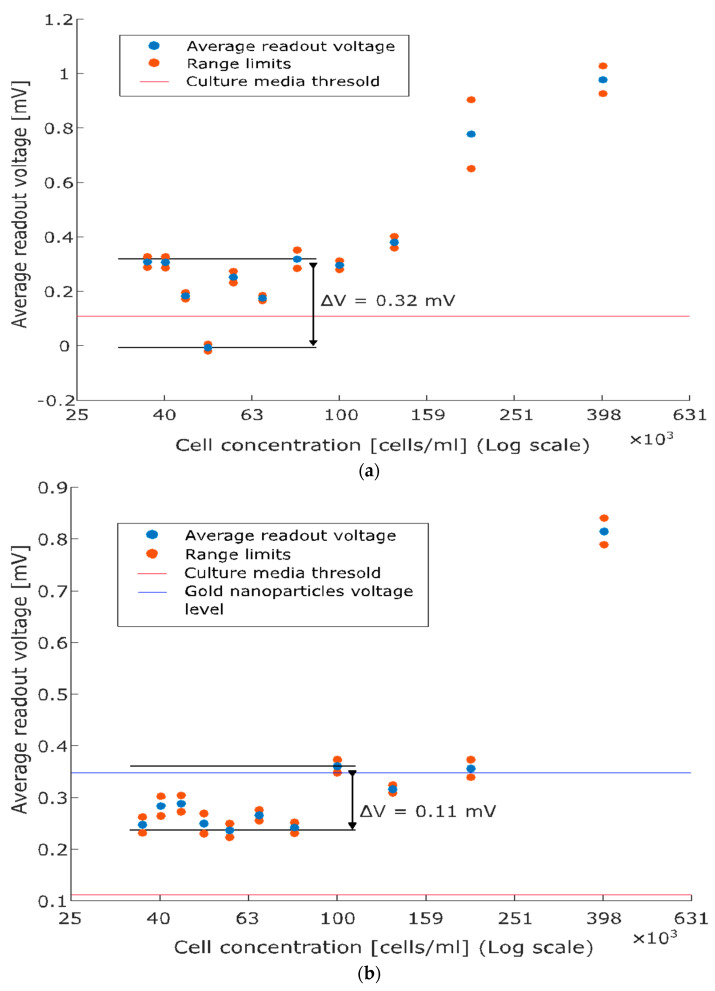
Average readout voltage versus cell concentration (calibration curves). (**a**) PC3: the monotonically increasing relationship holds at concentrations above 100 cells/mL while fluctuations are observed below. The baseline threshold is reported for comparison with the scatter plot. (**b**) Labelled PC3: gold nanoparticles (AuNPs) enhance capacitive detection, marking the monotonic character of the relationship and reducing the fluctuations at low concentrations. The contribution of solely AuNPs is also reported.

Finally, Figure 5 depicts the calibration curves for PC3 and LbPC3 (average readout voltages versus cell concentrations), which both monotonically increase at concentrations above 100 cells/mL. Fluctuations are observed at lower concentrations (sensitivity limitations), which are more evident with PC3 (Figure 5a) while slightly dampened, though not eliminated, with the AuNP labelling (Figure 5b).

### 4.2. Dynamic Characterization

To reduce the amount of the sample required and overcome limitations due to a rearrangement of the drop in standard environmental conditions (affected by temperature and humidity) and to achieve a more reproducible experimental environment, we set up a microfluidic platform, embedding the sensing module.

Figure 3b,c shows the second benchtop configuration, including the microfluidic channel, in which the fluids under test were injected by the Elveflow OB1 MK3+ microfluidic controller at low pressure (100 mbar) to simulate the spilling of the sample out of the bioreactor.

The readout voltage was sampled at time intervals ranging from 50 to 300 s, starting from less than five seconds after the beginning of the flow. Both PC3 and LbPC3 were tested at different concentrations. The cell culture was diluted until it was no longer detectable.

Firstly, human prostate cancer cells (PC3) were analyzed starting from a suspension of 100 µL of cells in DMEM. Concentrations of 4 and 2 × 10^5^ cells/mL were tested. Then, equal amounts of gold nanoparticle-labelled cells (LbPC3) were used. It is worth noting that the volume of liquid enclosed in the straight portion of the microfluidic channel (0.3 mm × 0.2 mm × 10 mm) is 600 nL, containing around 240 cells in the channel volume at the highest concentration considered. Finally, a 2 nM AuNP suspension in DMEM was tested.

**Figure 6:**
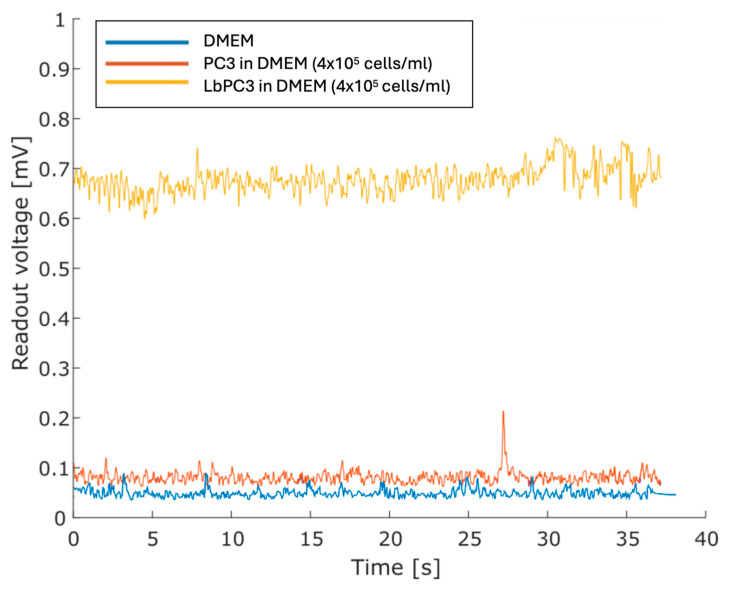
Measurements with the DMEM culture medium only and with PC3 and labelled PC3 (LbPC3) suspensions. The average readout voltage for the LbPC3 cell suspension was above the baseline, as expected.

Figure 6 displays the voltage traces. The cell-free DMEM culture medium was taken as the baseline. The highest PC3 cell suspension showed no significant displacement. For the concentrated LbPC3 suspension, on the other hand, the average voltage amplitude was significantly over the baseline.

**Figure 7:**
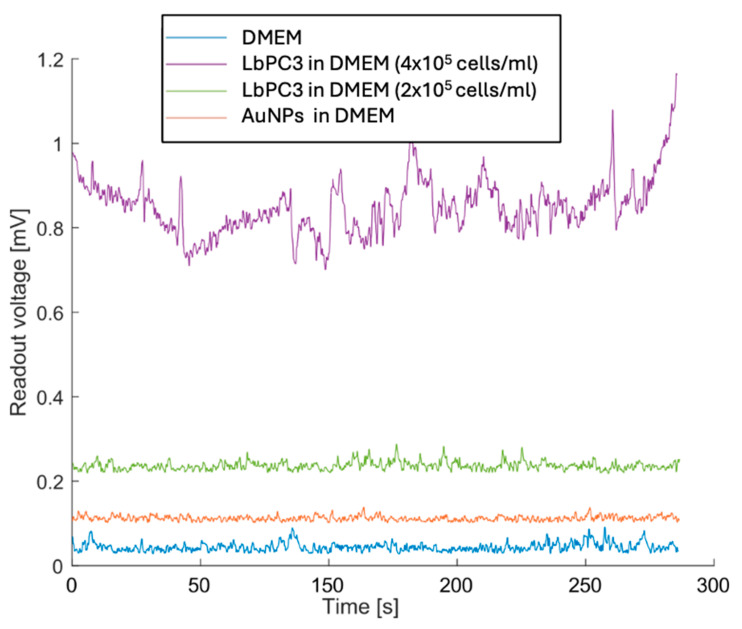
Experimental results with the most concentrated (400,000 LbPC3 in DMEM) and diluted labelled PC3 (200,000 LbPC3 in DMEM) cell suspensions (the second obviously yielded a voltage that was lower but still above the baseline). The gold nanoparticles (AuNPs) suspension in DMEM is clearly detected but less effective in raising the voltage.

Figure 7 reports the measurements with LbPC3 cells at the two different concentrations where, consistent with Figure 6, the average voltages are easily detectable and clearly distinct from each other. Lower concentrations were not detectable. It should be pointed out, however, that at the second dilution (2 × 10^5^ LbPC3/mL in DMEM), there were around 120 labelled cells in the microfluidic channel. A 10 nM AuNP suspension in DMEM was also tested to assess the contribution of gold nanoparticles alone. In this case, the signal was appreciably above the baseline, though lower than with particles grafted to the cells.

Finally, Table 1 reports the estimated cell counts in the microchannel. The absolute numbers of cells are obviously far smaller than in the drop experiment, being constrained by the small volume of the channel. The latter also reduces the size of the effective inter-electrode sensing area considerably. Yet, the labelled cells produce detectable voltage changes. This is a key remark in view of the final application, in which the fraction of cells subtracted from the population should be kept as small as possible.

As a manufacturing note, repeated testing tends to wear out the microfluidic module, which then becomes leaky, causing material residues to remain in the sensing area. For this reason, after iterated experiments, the sensors often exhibited unusual offsets (even in the 15 mV range), which made them unusable. After removing the microfluidics, the sensor returned to reliable measurements with no offset. This issue is related to the prototypal fabrication method and will not impact the final device.

## 5. Conclusions

Automatic cell counters often sort cells based on their size or their electrophysiological or mechanical features, enabling label-free classification even in heterogenous samples, also according to multiparametric approaches [25,26]. However, when the only distinctive feature of a subpopulation is the presence of a specific antigen on the membrane surface, this approach hardly yields significantly different readings and engineered CAR-T cells just differ from native T-lymphocytes because of the presence of the Chimeric Antigen Receptor (CAR) on their surface.

The LoC cell detector demonstrated here combines standard cell-specific gold nanoparticle labelling with a simple, low-cost capacitive detector. There is no need for complex evaluations at the single-cell level, which, on the other hand, would hardly reveal the key feature. Instead, this system extracts reliable statistics on a small number of cells. In addition, the present configuration could be easily integrated into a more complex microfluidic device, allowing not only the in-line labelling of cells but also the possibility for identifying different subsets of cells. With this aim, several kinds of nanoparticles, and, consequently, different transduction systems, are available, as well as microfluidic components that are able to handle the functionalization steps and sorting [19].

Again, LoCs often deploy complex architectures (e.g., including external AC sources, Electrochemical Impedance Spectroscopy, EIS, and Lock-In Amplifiers, LIAs, for electric measurements) to the detriment of industrialization [27,28,29,30]. The system described here is based on a commercial sensor, requiring neither external sources nor complex measurement apparatus. It is fully integrable on-chip, offering clear advantages in terms of simplicity, compactness, and cost-effectiveness, all of which are primary goals of the LoC approach. This setup overcomes current limitations in the industrial development of LOC devices, which often rely on complex architectures (such as external AC sources, Electrochemical Impedance Spectroscopy, EIS, and Lock-In Amplifiers, LIAs, for electric measurements) [27,28,29,30].

The device performances are suitable for real-world applications, given that the estimated number of cells in the 600 nL volume of the channel is small compared to the 10^7^–10^8^ CAR-T per kilogram of body weight [31] typically expanded inside bioreactors for patient reinfusion. 

Additionally, the power consumption is low (338.91 μW), meeting critical requirements for portable platforms.

Finally, while initially conceived for immunotherapy, this device could find application wherever cell subpopulations need to be sorted based on specific surface membrane characteristics.

## Figures and Tables

**Table 1:** Estimated labelled PC3 (LbPC3) cell counts and corresponding readout voltages. The DMEM baseline and gold nanoparticle (AuNP) suspension voltage are reported for comparison.

Target	Estimated Count	Average Readout Voltage
LbPC3 in DMEM (400,000 cells)	240 cells in the microchannel	0.89 mV
LbPC3 in DMEM (200,000 cells)	120 cells in the microchannel	0.25 mV
AuNPs in DMEM	-	0.1 mV
DMEM (baseline)	-	0.05 mV

## Data Availability

The data were fully exploited and described in the paper.

## Supplementary Materials

The following supporting information can be downloaded at: https://www.mdpi.com/article/10.3390/s24227329/s1. Figure S1: PC3 cells labelled with fluorescent gold nanoparticles; Figure S2: overview of the software; and Figure S3: characterization of the capacitive sensor.
