# Supplementary material for: A Microfluidic-Based Sensing Platform for Rapid Quality Control on Target Cells from Bioreactors
Source: Sensors (Basel). 2024 Nov 16;24(22):7329. doi: 10.3390/s24227329 (PMC11598262; doi:10.3390/s24227329)
Supplement: Supplementary file 1 [file sensors-24-07329-s001.zip › sensors-3301842-supplementary.pdf]

Supplementary Information

A Microfluidic-Based Sensing Platform for Rapid Quality Control on Target Cells from Bioreactors

Alessia Foscarini [1,2,†], Fabio Romano [3,†], Valeria Garzarelli [1,2,4], Antonio Turco [1], Alessandro Paolo Bramanti [3,5], Iolena Tarantini [1,2], Francesco Ferrara [1,*], Paolo Visconti [3], Giuseppe Gigli [1,2] and Maria Serena Chiriacò [1]

1 CNR Nanotec Institute of Nanotechnology, Via Monteroni, 73100 Lecce, Italy; alessia.foscarini@nanotec.cnr.it (A.F.); valeria.garzarelli@nanotec.cnr.it (V.G.); antonio.turco@nanotec.cnr.it (A.T.); iolena.tarantini@unisalento.it (I.T.); giuseppe.gigli@unisalento.it (G.G.); mariaserena.chiriaco@nanotec.cnr.it (M.S.C.)

2 Department of Experimental Medicine, University of Salento, 73100 Lecce, Italy

3 Department of Innovation Engineering, University of Salento, Via per Monteroni, Building 'O', 73100 Lecce, Italy; fabioromano9198@gmail.com (F.R.); alessandro.bramanti@st.com (A.P.B.); paolo.visconti@unisalento.it (P.V.)

4 Department of Mathematics and Physics, E. De Giorgi, University of Salento, Via per Arnesano, 73100 Lecce, Italy

5 STMicroelectronics srl, c/o Campus Ecotekne, Via per Monteroni, 165, 73100 Lecce, Italy

* Correspondence: francesco.ferrara@nanotec.cnr.it; Tel.: +39-0832-319120

† These authors contributed equally to the work.

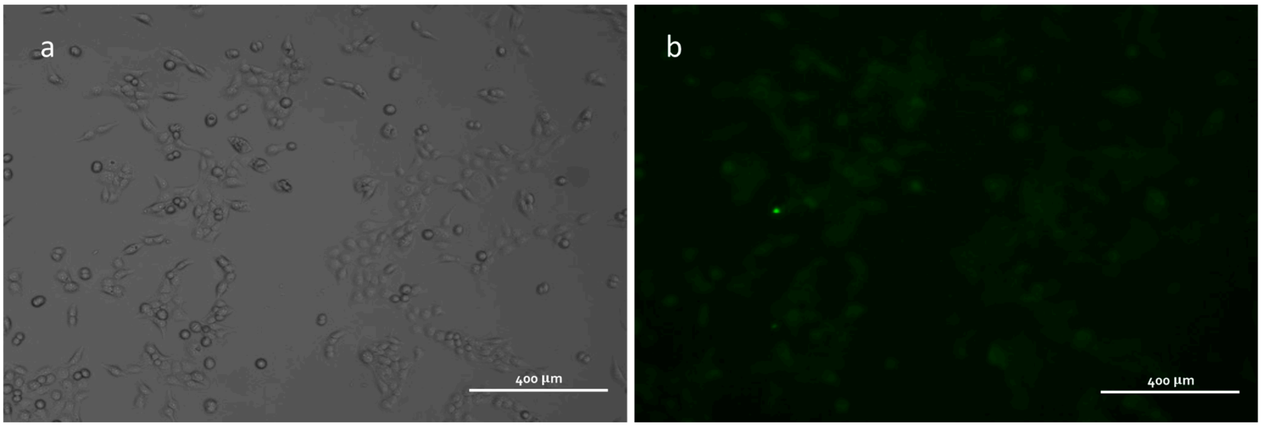

Supplementary Figure S1. PC3 cells labelled with fluorescent golds nanoparticles. (**a**) Bright-field optical microscope acquisition of PC3 cells cultured in adhesion. (**b**) Green fluorescence optical acquisition of label PC3 cells with gold nanoparticles functionalized with secondary FITC-conjugated antibody recognizing anti-EpCAM antibodies on cell membranes.

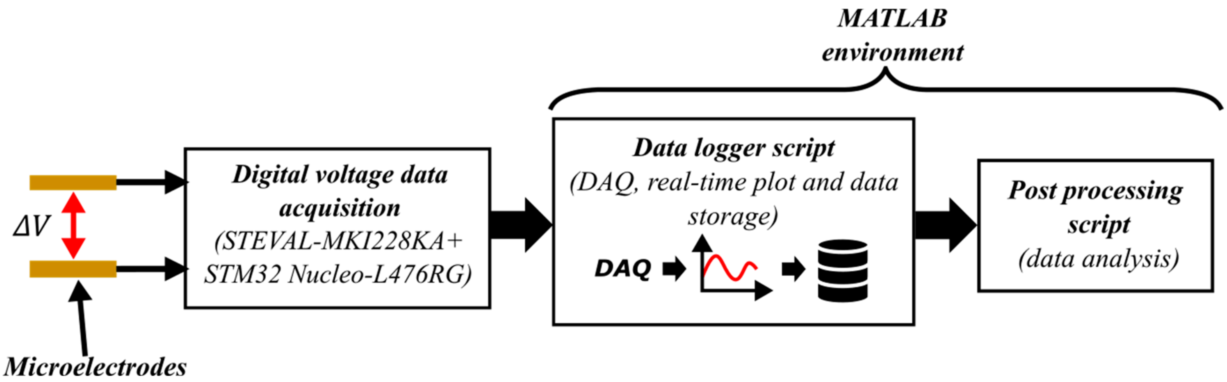

Supplementary Figure S2. Overview of the software. The firmware acquires the data passing it to the user interface for further processing.

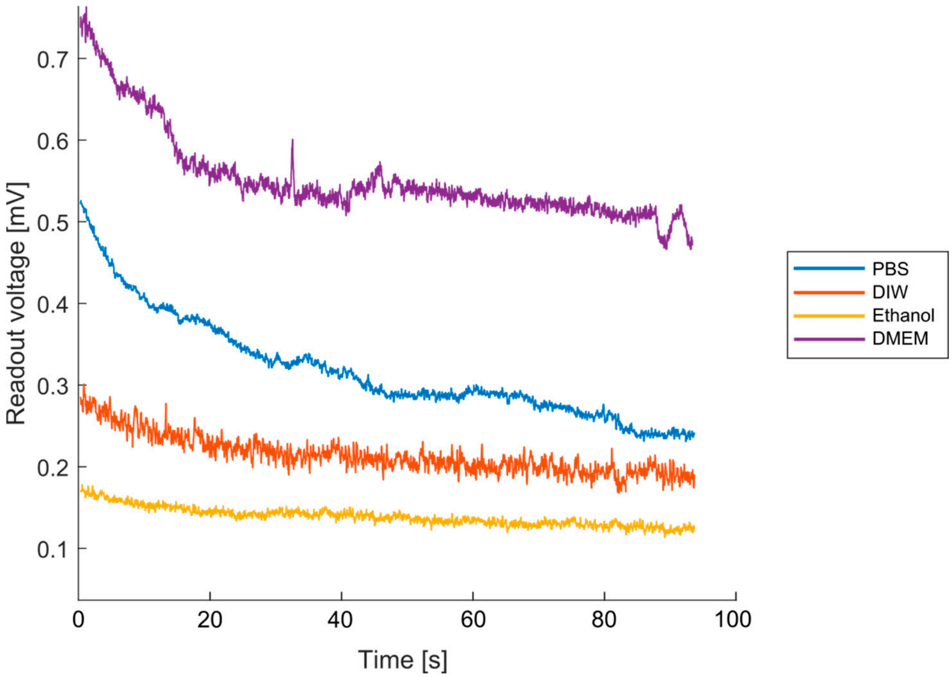

Supplementary Figure S3. Characterization of the capacitive sensor. Capacitive response to different fluids commonly used for cell culture: phosphate-buffered saline (PBS), deionized water (DIW), ethanol and Dulbecco's modified eagle medium (DMEM).
